# Compensatory effects of serotonin on the regulation of blood volume in healthy human subjects

**DOI:** 10.14814/phy2.70620

**Published:** 2025-10-22

**Authors:** Yuki Sakai, Hideya Momose, Tomomi Watanabe‐Asaka, Moyuru Hayashi, Nariaki Arai, Norika Kuneshita, Daisuke Maejima, Nau Ishimine, Yoshiko Kawai, Toshio Ohhashi

**Affiliations:** ^1^ Department of Innovation of Medical and Health Sciences Research Shinshu University School of Medicine Matsumoto Japan; ^2^ Department of Dentistry and Oral Surgery Shinshu University School of Medicine Matsumoto Japan; ^3^ Division of Physiology, Faculty of Medicine Tohoku Medical and Pharmaceutical University Sendai Japan; ^4^ Department of Anesthesiology and Resuscitology Shinshu University School of Medicine Matsumoto Japan; ^5^ Department of Laboratory Medicine Shinshu University Hospital Matsumoto Japan

**Keywords:** blood volume, human, physical exercise, serotonin, vasopressin

## Abstract

It has been demonstrated that water intake accelerates the release of serotonin from enterochromaffin cells in the rat jejunum, which is then transported through the portal vein into the blood. However, the physiological roles of serotonin in the regulation of blood volume remain unclear. We aimed to investigate the roles of serotonin in the physiological regulation of physical exercise‐dependent sweating‐mediated haemoconcentration in humans. All participants performed two times 30‐min sessions of ‐up‐and‐down physical exercise with a 20‐min rest. Changes in thermal perspiration rate, concentrations of serotonin and vasopressin in blood, and samples of blood and urine were measured just before and after repeated exercise. All participants presented increased vasopressin after the first trial but showed no change after the second exercise session. In contrast, blood serotonin levels increased dramatically after the second session. Urine osmolality increased significantly after the first exercise session. All the participants showed significantly decreased ratios of red blood cells, hemoglobin, and neutrophils before and after the second exercise. In contrast, no significant changes were observed in the ratios of total protein and lymphocytes. In conclusion, serotonin may play a crucial role in the compensatory regulation of sweating‐mediated haemoconcentration in cooperation with vasopressin.

## 1 INTRODUCTION

We previously developed a system that informs users of the risk of heatstroke through a wearable perspiration rate meter and by monitoring vasopressin‐related thirst response (Momose et al., 2023). To validate the system, we conducted 30‐min step‐up and step‐down physical exercises in humans (Momose et al., 2023). The exercise strength was approximately 70 nm, which is moderate level, and the average pulse rates increased by 120–130 beats/min. We also investigated the relationships between sweating‐mediated total water loss and induced sweating‐mediated water loss in the body (Dubois & Dubois, 1916; Momose et al., 2023). However, the compensatory regulatory mechanisms of perspiration‐mediated haemoconcentration in human subjects have not been fully clarified.

On the other hand, serotonin (5‐hydroxytryptamine; 5‐HT) is known to be predominantly produced in the small intestine, being approximately 95% of the body's serotonin (Gershon, 2013; Brummelte, 2017; Kajihara et al., 2021; Terry & Margolis, 2017). 5‐HT is mainly stored in enterochromaffin cells (EC) and nerve terminals of the intestine (Ahlman, 1983). Vagal nerve stimulation releases 5‐HT from the EC through a β‐adrenoceptor mechanism of the included sympathetic adrenergic fibers (Terry & Margolis, 2017). Additionally, serotonin is known to modulate dehydration‐induced changes in bitter water tolerance in human subjects (Iwai et al., 2015).

Previous studies demonstrated that water intake accelerates the release of serotonin from the EC in rat jejunal villi, which is transported through the portal vein into the blood circulation, but not into the mesenteric lymph circulation, as serotonin is easily conjugated with stored long‐chain fatty acids in the jejunal villi (Kajihara et al., 2021). These findings are consistent with the evidence that rat mesenteric lymphatic smooth muscles are highly sensitive to 5‐HT, resulting in spastic contraction with10^−6^ M 5‐HT, which inhibits mesenteric lymph transport (Ohhashi et al., 2005). In contrast, increased serotonin in the blood stimulates lymph formation in the jejunal villi by accelerating the permeability of plasma albumin through venular endothelial cells in the jejunal microcirculation (Amari et al., 2022; Guyton & Taylor, 1975; Hayashi et al., 2020), which contributes to accelerating the transport of water‐soluble small substances from the interstitial tissues of the jejunal villi into the mesenteric lymph vessels (Amari et al., 2022). Therefore, 5‐HT may be a crucial regulator in mesenteric lymph formation and transport (Amari et al., 2022).

In addition, large amounts of mesenteric lymph produced hemodilution via the activation of lymph flow through the thoracic duct in humans (Kawai et al., 2015; Ohhashi & Sakaguchi, 1998). However, the regulatory roles of 5‐HT in blood volume in human subjects are still unknown. Based on the findings, we hypothesized that the serotonin released in the jejunum plays a compensatory physiological regulator for blood volume in cooperation with the release of vasopressin.

Furthermore, we also demonstrated that abdominal respiration during the rest in the supine position is effective for inducing lymph drainage in the abdominal and lower extremities, which induces hemodilution and lowering of blood vasopressin concentration (Kawai et al., 2015; Ohhashi & Sakaguchi, 1998). To evaluate the hypothesis, we examined the relationship between haemoconcentration produced by two repeated 30‐min sessions of physical exercise and changes in the concentrations of blood serotonin and vasopressin in healthy human participants.

## 2 MATERIALS AND METHODS

### 2.1 Ethical approval

This study was approved by the Ethics Committee for Human Clinical Studies at the School of Medicine, Shinshu University (CRB3200010, approval no. 4445, 6th August 2019). All study procedures were performed in accordance with the principles outlined in the Declaration of Helsinki. The study was registered on the WHO International Clinical Trial Registry Platform (13th/August/2022, https://www.who.int/clinical‐trials‐registry‐platform:jRCT1032220270).

### 2.2 Wearable perspiration ratemeter and self‐information apparatus with a smart phone

We have previously developed a highly sensitive perspiration suitable for measuring active palmar sweating (Knox, 1983). By modifying the original palmar perspiration ratemeter, we constructed a wearable, precise rate‐meter equipped with a capacitive humidity sensor (Momose et al., 2023). In the present study, we measured thermal sweating of the neck and thirst sensation using a wearable apparatus (SKINOS Co. LTD, Ueda, Nagano, Japan) in human participants with two repetitions of 30 min physical exercise. The skin surface area of the neck was kept at 1 cm^2^ to measure the total water loss in the body (Momose et al., 2023).

### 2.3 Human participants

In total, 13 healthy participants (mean age: 40.9 ± 26.3 years; six males and seven females) were enrolled in the present study. Minimized sample size was recommended by the ethics committee for the human observational studies. All the participants provided written and oral informed consent after receiving a detailed explanation of the human experiments. Each participant signed an informed consent document. All procedures were performed in accordance with the tenets of the Declaration of Helsinki. The obtained data were stored at Shinshu University School of Medicine. The grouping of participants according to the ethical description followed the reference (Momose et al., 2023).

All human experiments were conducted in the afternoon from 13:00 to 15:00, considering the maximal and stable sympathetic nerve activity in the human circadian rhythm. The protocol has been previously described (Momose et al., 2023). The examination room was maintained within the range of 24°C–26°C and 20%–30% humidity, respectively, using air conditioners.

### 2.4 Experimental protocols

A total of 16 healthy participants were initially enrolled in this study. However, three participants reported a disturbance in their physical condition and mental stress during the experiment. Thus, experimental data were analyzed for 13 participants.

**FIGURE 1:**
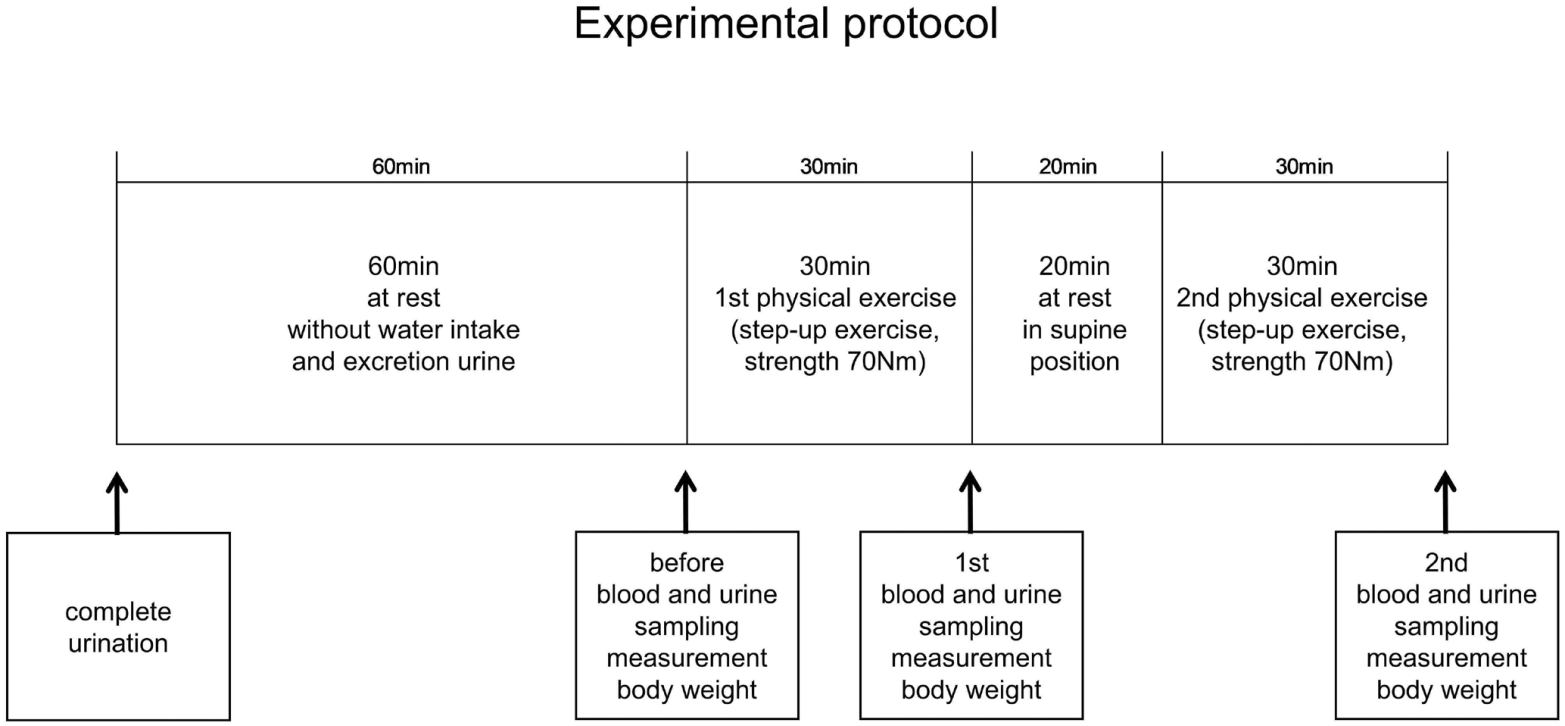
The schematic layout of experimental protocol. A total of 13 healthy human participants were enrolled in this study.

Figure 1 presents the schematic layout of the experimental protocol. The participants were instructed to empty their bladder completely and were restricted from water intake and urination for 1 h to the start of experiments. Blood and urine samples were collected immediately before the 30 min step‐up and step‐down (20 cm height) exercise. The strength of physical exercise was approximately 70 nm (a moderate level of physical exercise) in each exercise, and the average heart rates during the 30 min physical exercise increased by 121.3 ± 6.2 (after first exercise) and 130.1 ± 5.7 (after second exercise) beats/min (*n* = 13). In addition, two repetitions of 30 min physical exercise were performed after 20 min rest in the supine position between each exercise (Kawai et al., 2015; Ohhashi & Sakaguchi, 1998). Blood and urine samples were collected from all participants immediately after each physical exercise session.

To evaluate thermal sweating‐mediated haemoconcentration and its‐dependent physiological compensatory responses, the concentrations of total protein (TP), red blood cells (RBC), hemoglobin (Hb), lymphocytes (LYM), and neutrophils (NUT) in blood samples were measured by the Department of Laboratory Medicine at Shinshu University Hospital. The changes in urine volume and urine osmolarity of the subjects were analyzed in the same laboratory. In addition, changes in the concentrations of vasopressin and serotonin were measured just before and after the first and second trial using a serotonin assay kit with a serotonin polyclonal antibody (SRL Co. Inc., Tokyo, Japan). The changes in the concentrations of vasopressin were also measured with a vasopressin polyclonal antibody in the same company (SRL Co. Inc., Tokyo, Japan).

The total sweat volume, total water loss, changes in body weight, and thirst response were also investigated after 30 min of first and second physical exercise. To measure the water loss per body surface area, we used the formula 71.84 × height ^0.725^ × weight ^0.425^ × 10^−4^ for calculating body surface area (Dubois & Dubois, 1916).

### 2.5 Statistical analysis

All data were presented as mean ± standard deviation (SD). Statistical significance was analyzed using the Student's *t*‐test for unpaired observations after checking that the data conformed to the test distribution or one‐way ANOVA followed by Bonferroni correction, as appropriate. *p* < 0.05 was considered statistically significant.

## 3 RESULTS

### 3.1 Similar amounts of perspiration with repeated 30 min physical exercise

**FIGURE 2:**
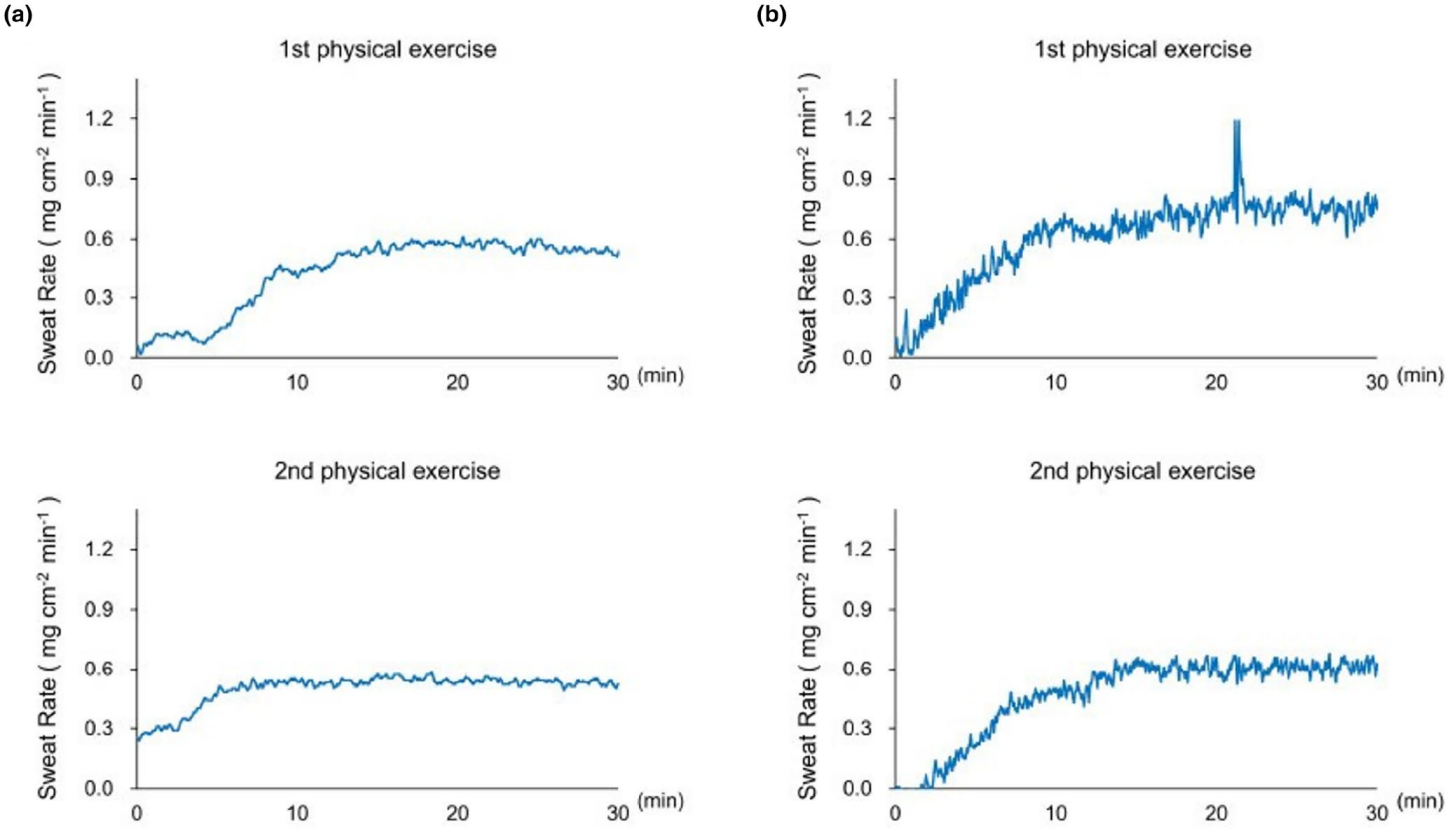
Representative physical exercise‐induced sweating curves measured using the wearable perspiration ratemeter at each neck. (a) 20‐year‐old female and (b); 38‐year‐old male. They had accepted each sign of the informed consent of humans after receiving a detailed explanation of the human experiments.

Figure 2 presents the representative thermal sweating curves measured on the necks of two participants ((a) 20‐year‐old female and (b) 38‐year‐old male) during two 30‐min bouts of physical exercises, with a 20‐min rest period in the supine position between the sessions. The perspiration curves of the two participants recorded in the first trial seemed to be almost similar to those in the second trial (Figure 2a, b). As shown in Figure 2b, the latent time for the initiation of perspiration tended to be shorter in the second exercise (latent time with the second trial; 3–5 min vs. 0.5–3 min).

### 3.2 No significant change in sweating rate and total water loss with repeated physical exercise

**FIGURE 3:**
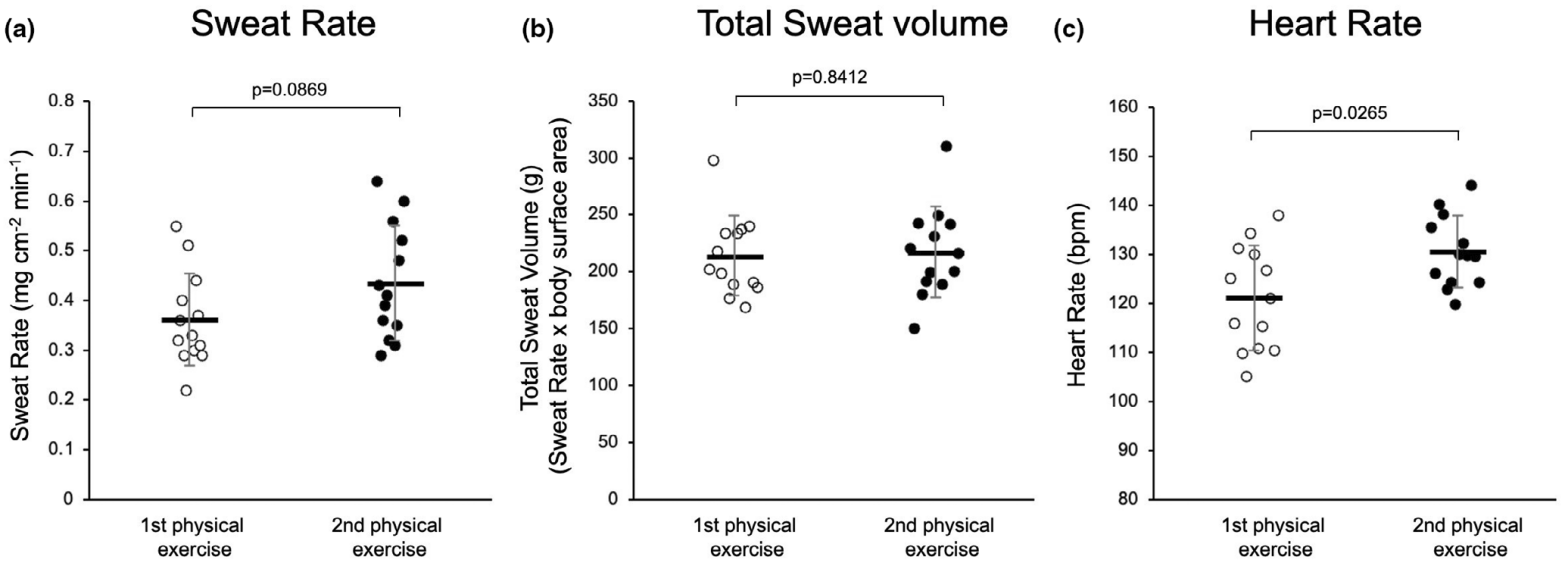
The summarized data of the sweating rate (mg/(cm^2^･min), (a)) and total water loss (sweating rate × body surface area, g (b)), and heart rate (c) of the all participants with repeated 30 min physical exercise.

Figure 3a presents the summarized data of the sweating rate of all participants with repeated 30‐min physical exercise (after the first and second exercise were 0.36 ± 0.10 and 0.44 ± 0.12 mg/cm^2^ min, respectively, *p* = 0.0869, *n* = 13). Figure 3b shows the total water loss, sweating rate × body surface area (after first exercise 213.3 ± 35.1 g and after second exercise 217.0 ± 39.8 g, respectively, *p* = 0.8412, *n* = 13).

### 3.3 Significant increase in heart rate after second physical exercise

Figure 3c demonstrates the changes in heart rate with the repeated exercise. The heart rate after the second exercise is significantly increased compared with that after the first exercise (121.0 ± 10.6 beats/min after the first exercise vs. 130.5 ± 7.3 beats/min after the second exercise, respectively, *p* = 0.0285, *n* = 13).

### 3.4 Vasopressin concentration significantly increased after first exercise but not after the second exercise

**FIGURE 4:**
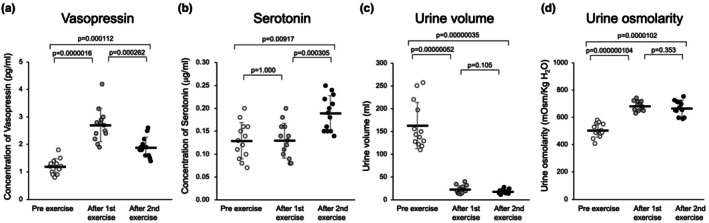
(a) The changes in blood vasopressin of pre‐exercise, after first exercise and after second exercise. (b) The changes in serotonin concentrations before and after the first and second exercise. (c) Summarized data of urine volume (mL) before and after the repeated physical exercise. (d) Summarized data of urine osmolarity (mOsm/kg H_2_O) before and after the repeated physical exercise.

Figure 4a shows the changes in vasopressin concentrations immediately before and after the 30‐min first and second physical exercise in all participants (*n* = 13). The blood vasopressin concentration after the first exercise was significantly higher compared with the concentration before the exercise (1.2 ± 0.2 pg/mL pre‐exercise vs. 2.7 ± 0.6 pg/mL after first post‐exercise, *p* = 0.0000016, *n* = 13). All the participants showed a significant increase in vasopressin concentration after the first exercise compared with that after the second exercise (2.7 ± 0.6 pg/mL after the first exercise vs. 1.9 ± 0.4 pg/mL after the second exercise, *p* = 0.000262, *n* = 13).

### 3.5 Serotonin concentration significantly increased after the second exercise

In contrast, serotonin concentration significantly increased after the second exercise compared with that after the first exercise. Figure 4b presents the summarized data of serotonin concentration after the first and after second exercise in all participants (just before exercise 0.13 ± 0.04 μg/mL; after first exercise 0.13 ± 0.04 vs. after second exercise 0.19 ± 0.04 μg/mL; the first vs. second *p* = 0.000305 *n* = 13; just before exercise vs. after first exercise, *p* = 1.000 *n* = 13).

### 3.6 Urine volume significantly decreased both after the first and after second exercise

Figure 4c shows the summarized data of urine volume just before, after the first, and after the second exercise in all participants (just before 163.4 ± 51.1 mL, after first exercise 23.1 ± 8.6 mL, and after second exercise 18.5 ± 4.8 mL; just before vs. after first exercise, *p* = 0.00000052 *n* = 13; after first exercise vs. after second exercise *p* = 0.105, *n* = 13).

### 3.7 Urine osmolarity significantly increased both after the first and after second exercise

Figure 4d shows the changes in urine osmolality in all participants (*n* = 13) immediately before and after 30‐min first and second bouts of physical exercise. Urine osmolarity increased significantly after the first and after the second exercise (just before 504.5 ± 50.3 mOsm/kg H_2_O mL, after first exercise 686.1 ± 35.2 mOsm/kg H_2_O mL, and after second exercise 671.0 ± 53.7 mOsm/kg H_2_O mL; just before vs. after first exercise, *p* = 0.000000104, *n* = 13; after first exercise vs. after second exercise, *p* = 0.353, *n* = 13).

### 3.8 Changes in the ratio of blood concentrations of TP, RBC, Hb, LYM, and NUT after the first and after second exercise to those measured before exercise

**FIGURE 5:**
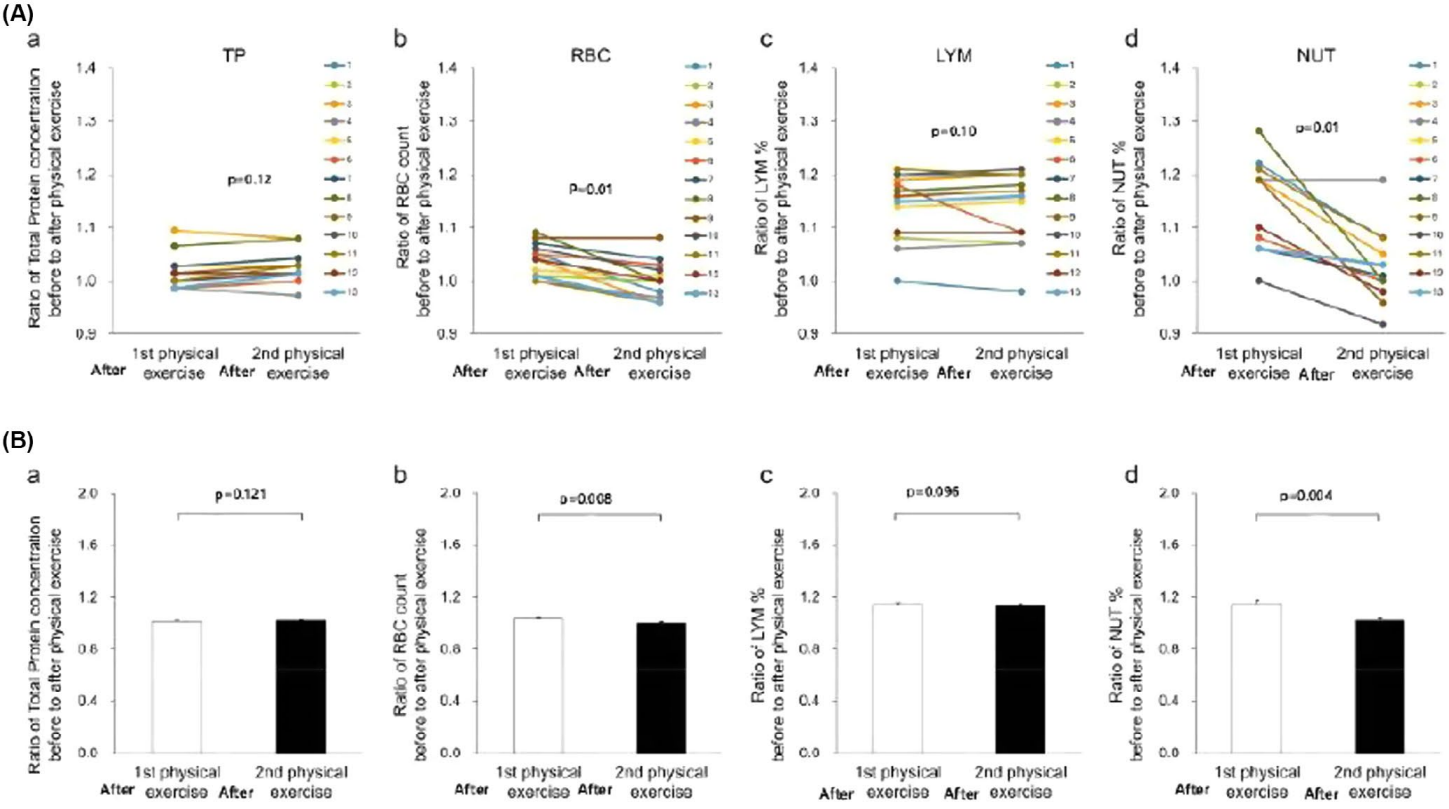
(A) Representative recordings of changes in the ratios of the blood concentrations of TP (a), RBC (b), LYM (c), and NUT (d) of all participants before and after the repeated physical exercise. (B) The summarized data for the ratios of the blood concentrations of TP (a), RBC (b), LYM (c), and NUT (d) of all participants before and after the repeated exercise.

Figure 5A presents the representative changes in the ratios before and after 30 min of the first and second physical exercise concerning the blood concentrations of TP (Figure 5A‐a), RBC (Figure 5A‐b), LYM (Figure 5A‐c), and NUT (Figure 5A‐d) in all participants (*n* = 13). Figure 4b shows the summarized data.

The ratio of TP before and after 30‐min first and second of physical exercise showed little change (after first exercise 1.02 ± 0.07% vs. after second exercise 1.03 ± 0.14%, *p* = 0.121, *n* = 13). Similar to the change of TP, the ratio of LYM after the first exercise to after the second shows no significant change (after first exercise 1.14 ± 0.14% vs. after second exercise 1.14 ± 0.14%, *p* = 0.096, *n* = 13).

In contrast, the ratios of RBC and NUT before and after the second exercise significantly decreased in all participants (RBC; after first exercise 1.04 ± 0.07% vs. after second exercise 1.00 ± 0.07%, *p* = 0.008, *n* = 13 vs. NUT; after first exercise 1.15 ± 0.22% vs. after second exercise 1.02 ± 0.22%, *p* = 0.004, *n* = 13). Similar to the change in RBC, the ratio of Hb concentration before and after the second exercise decreased in all participants (after first exercise 1.01 ± 0.11% vs. after second exercise, 0.98 ± 0.14%, *p* = 0.0009, *n* = 13).

## 4 DISCUSSION

This study aimed to investigate the potential roles of serotonin released from the jejunum in the physiological regulation of physical exercise‐dependent sweating‐mediated haemoconcentration in humans. We examined the blood serotonin and vasopressin concentrations in repeated bouts of exercise in healthy humans and found the different responses, which suggested some relationships to the balance between sweat‐induced haemoconcentration and lymph‐mediated hemodilution.

In the repeated 30‐min interval physical exercise with 20‐min rest in the supine position, the similar sweating rates were observed with the first and second exercises in all participants. All participants showed significantly increased plasma vasopressin concentrations after the first exercise compared with those after the second exercise. These results suggested the increase of blood NaCl concentration‐mediated vasopressin release from the pituitary gland. Vasopressin may be produced by the increase in total sweat volume (Figure 2). Thus, the vasopressin concentration in all participants significantly increased after the first 30‐min exercise and decreased after the second exercise. This increase in blood volume after the first exercise may be related to the vasopressin‐mediated rapid increase in urine reabsorption. However, the increase in urine osmolarity continued after the second exercise, which may be related to a continuous decrease in urine volume. The long‐period action of vasopressin on urine reabsorption in the kidney may also contribute to the increases in urine osmolarity and the decrease in urine volume. The findings may be in part related to the hemodilution after the second exercise, resulting in the decrease of vasopressin in blood.

The ratios of RBC, Hb, and NUT before and after physical exercise significantly decreased after the second exercise. However, no significant changes were observed in the ratios of TP and LYM immediately before and after the physical exercise. Similar changes in the concentrations of albumin (Alb), hematocrit (Hct), Hb, white blood cells, and platelets were confirmed with abdominal respiration‐mediated hemodilution in human participants in a previous study (Momose et al., 2023). The 30‐min abdominal respiration in the supine position caused more significant hemodilution, with significant reductions of TP, Alb, RBC, Ht, and ADH levels in all participants (Momose et al., 2023). In fact, the ratios of RBC, Hb, and NUT before and after the second exercise significantly decreased in all participants in this study. These marked changes indicated hemodilution, which may have contributed to the decrease in blood vasopressin concentrations after the second trial (Figure 3a). The repeated 30‐min physical exercise with 20‐min rest in the supine position may accelerate lymph flow through the thoracic duct, enhancing hemodilution (Kawai et al., 2015; Ohhashi & Sakaguchi, 1998). The lymph flow rate through the lymphatic system is very slow (Ohhashi et al., 2005), which may also be related to the acceleration of the hemodilution after the second exercise.

In addition, the 1.5 –3.0 g/mL amounts of albumin contained in the lymph (Knox, 1983; Ohhashi et al., 2005) may be related to no significant change in the ratio of TP before and after repeated physical exercise (Figure 5A‐a, 5B‐a). The lack of change in lymphocyte concentration after the first and second exercise may be related to the fact that lymphocyte excretion from regional lymph nodes is dependent on the concentration of Alb in the afferent lymph vessels (Knox, 1983).

Interestingly, blood serotonin concentration increased dramatically after the second exercise, with little or no change after the first exercise. In terms of the changes in the ratios of serotonin concentration in the blood before and after repeated physical exercise, the reduction of vasopressin release‐mediated hemodilution after the second exercise may emphasize the increased blood serotonin concentration in all participants (Figure 3b). It is well known that physical exercise accelerates serotonin release from the small intestine via sympathetic activation (Ahlman, 1983; Lundgren, 2002). Thus, the reasons why all participants showed increased blood serotonin concentration after the second trial may be, in part, related to the excitation of nerve fibers‐mediated slow release of serotonin from the small intestine and slow transport of serotonin through the portal vein and liver (Ahlman, 1983; Iwai et al., 2015). In addition, the changes in the concentration of serotonin in blood may not be clarified in the strong vasopressin‐mediated hemodilution after the first exercise.

In conclusion, serotonin released from the small intestine may play a crucial role in the compensatory regulation of physical exercise‐mediated haemoconcentration in cooperation with vasopressin and the thirst response. However, to confirm the conclusion, future studies will be needed to continuously monitor changes in plasma osmolarity and plasma volume.

## AUTHOR CONTRIBUTIONS

Toshio Ohhashi, Yuki Sakai, Hideya Momose, and Yoshiko Kawai designed the experiments, conducted the experiments, analyzed the data, constructed the figures, and wrote the original manuscript. Tomomi Watanabe‐Asaka, Moyuru Hayashi, Nariaki Arai, Daisuke Maejima, Norika Kuneshita, and Nau Ishimine conducted the experiments, analyzed the data, and revised the manuscript All.

## FUNDING INFORMATION

The Department of Innovation of Medical and Health Sciences Research at Shinshu University School of Medicine has been established and supported financially by the donation of BOURBON, Co., Ltd., Kashiwazaki, Niigata, Japan, and Aizawa Hospital, Matsumoto, Nagano, Japan. The authors declare that this study received funding from BOURBON Co. Ltd. The funder was not involved in the study design, collection, analysis, interpretation of data, the writing of this article, or the decision to submit it for publication.

## CONFLICT OF INTEREST STATEMENT

No conflicts of interest, financial or otherwise, are declared by the authors.

## ETHICS STATEMENT

The study was approved by the Ethical Committee for Human Clinical Studies at the School of Medicine, Shinshu University (CRB3200010, approval no. 4445 on 6th August 2019). The study was registered in the WHO International Clinical Trial Registry Platform (13th/August/2022, https://www.who.int/clinical‐trials‐registry‐platform:jRCT1032220270).

## CONSENT TO PARTICIPATE

All participants provided written and oral informed consent after the detailed explanation of the human experiments. All participants accepted the informed consent.

## CONSENT FOR PUBLICATION

All authors approved the final version of the manuscript and accepted it for the publication of this manuscript.

## Data Availability

All relevant data are available from the corresponding author on request.

## References

[phy270620-bib-0001] Ahlman, H. (1983). Dahlstrom a vagal mechanism controlling serotonin release from the gastrointestinal tract and pyloric motor function. Journal of the Autonomic Nervous System, 9, 119–140. 6198349 10.1016/0165-1838(83)90136-4

[phy270620-bib-0002] Amari, K. , Kajihara, R. , Arai, N. , Hayashi, M. , Watanabe‐Asaka, T. , Kaidoh, M. , Yokoyama, Y. , Ajima, K. , Maejima, D. , & Kawai, Y. (2022). Ohhashi T portal blood flow‐dependent NO‐mediated lymph formation in rat jejunum. Pflügers Archiv‐European Journal of Physiology, 474, 541–551. 35157133 10.1007/s00424-022-02670-2

[phy270620-bib-0008] Brummelte, S. (2017). Introduction: Early adversity and brain development. Neuroscience, 342, 1–3. 27670901 10.1016/j.neuroscience.2016.09.041

[phy270620-bib-0003] Dubois, D. , & Dubois, E. F. (1916). A formula to estimate the approximate surface area if height and weight be known. Archives of Internal Medicine, 17, 863–871.

[phy270620-bib-0004] Gershon, M. D. (2013). 5‐Hydroxytryptaime in the gastrointestinal tract. Current Opinion in Endocrinology, Diabetes and Obesity, 20, 14–21. 23222853 10.1097/MED.0b013e32835bc703 PMC3708472

[phy270620-bib-0006] Guyton, A. C. , & Taylor, A. (1975). Grange HJ circulatory physiology II (pp. 125–160). Dynamics and Control of Body Fluids. Philadelphia.

[phy270620-bib-0007] Hayashi, M. , Watanabe‐Asaka, T. , Maejima, D. , Nagashio, S. , Kajihara, R. , Amari, K. , Yokoyama, Y. , Kaidoh, M. , Sugano, M. , Honda, T. , & Kawai, Y. (2020). Ohhashi T evaluating lymph flow through the thoracic duct using urine osmolarity in human subjects. Lymphatic Research and Biology, 18, 351–359. 31904309 10.1089/lrb.2019.0054

[phy270620-bib-0009] Iwai, M. , Muroi, K. , & Kinoshita, T. (2015). Ishii I serotonin modulates the dehydration‐induced changes in tolerance for bitter water. Physiology & Behavior, 151, 545–550. 26325013 10.1016/j.physbeh.2015.08.034

[phy270620-bib-0010] Kajihara, R. , Amari, K. , Arai, N. , Nagashio, S. , Hayashi, M. , Watanabe‐Asaka, T. , Kaidoh, M. , Yokoyama, Y. , Maejima, D. , & Kawai, Y. (2021). Ohhashi T water intake releases serotonin from enterochromaffin cells in rats jejunal villi. Pflügers Archiv‐European Journal of Physiology, 473, 921–936. 33913004 10.1007/s00424-021-02569-4

[phy270620-bib-0011] Kawai, Y. , Ajima, K. , Nagai, T. , Yokoyama, Y. , Kaidoh, M. , Seto, E. , & Honda, T. (2015). Ohhashi T abdominal respiration induces hemodilution and related reduction in ADH concentration. Lymphatic Research and Biology, 13, 202–207. 26305375 10.1089/lrb.2015.0017

[phy270620-bib-0012] Knox, P. (1983). Pflug J J the effect of canine popliteal node on flow composition of lymph. Journal of Physiology (London), 345, 1–14. 6663494 10.1113/jphysiol.1983.sp014961 PMC1193780

[phy270620-bib-0013] Lundgren, M. (2002). Sympathetic input into the enteric nervous system. Gut (Suppl IV) 47, 33–35. 10.1136/gut.47.suppl_4.iv33 PMC1766807 11076905

[phy270620-bib-0014] Momose, H. , Takasaka, M. , Watanabe‐Asaka, T. , Hayashi, M. , Maejima, D. , & Kawai, Y. (2023). Ohhashi T heatstroke risk informing system using wearable perspiration ratemeter on users undergoing physical exercise. Scientific Reports, 13, 419. 36624139 10.1038/s41598-023-27492-9 PMC9829658

[phy270620-bib-0015] Ohhashi, T. , Mizuno, R. , & Ikomi, F. (2005). Kawai Y current topics of physiology and pharmacology in the lymphatic system. Pharmacology & Therapeutics, 106, 165–188. 10.1016/j.pharmthera.2004.10.009 15670625

[phy270620-bib-0016] Ohhashi, T. , & Sakaguchi, M. (1998). Tsuda T Human perspiration measurement. Physiological Measurement, 19, 449–461. 9863672 10.1088/0967-3334/19/4/001

[phy270620-bib-0017] Terry, N. , & Margolis, K. G. (2017). Serotonergic mechanisms regulating the GI tract: Experimental evidence and therapeutic relevance. Handbook of Experimental Pharmacology, 239, 319–342. 28035530 10.1007/164_2016_103 PMC5526216

